# The flexibility and dynamics of protein disulfide isomerase

**DOI:** 10.1002/prot.25159

**Published:** 2016-10-01

**Authors:** Rudolf A. Römer, Stephen A. Wells, J. Emilio Jimenez‐Roldan, Moitrayee Bhattacharyya, Saraswathi Vishweshwara, Robert B. Freedman

**Affiliations:** ^1^ Department of Physics and Centre for Scientific ComputingThe University of WarwickCoventryCV4 7ALUnited Kingdom; ^2^ Department of Chemical EngineeringUniversity of BathBathBA2 7AYUnited Kingdom; ^3^ Molecular Biophysics Unit, Indian Institute of ScienceBangalore560012India; ^4^ School of Life SciencesThe University of WarwickCoventryCV4 7ALUnited Kingdom; ^5^ Present address: Moitrayee Bhattacharyya's current address is Department of Molecular and Cell BiologyUniversity of California BerkeleyCalifornia94720.

**Keywords:** PDI, protein flexibility, protein dynamics, large‐domain motion, dynamics of biomolecules, theory, modeling, and computer simulation

## Abstract

We have studied the mobility of the multidomain folding catalyst, protein disulfide isomerase (PDI), by a coarse‐graining approach based on flexibility. We analyze our simulations of yeast PDI (yPDI) using measures of backbone movement, relative positions and orientations of domains, and distances between functional sites. We find that there is interdomain flexibility at every interdomain junction but these show very different characteristics. The extent of interdomain flexibility is such that yPDI's two active sites can approach much more closely than is found in crystal structures—and indeed hinge motion to bring these sites into proximity is the lowest energy normal mode of motion of the protein. The flexibility predicted for yPDI (based on one structure) includes the other known conformation of yPDI and is consistent with (i) the mobility observed experimentally for mammalian PDI and (ii) molecular dynamics. We also observe intradomain flexibility and clear differences between the domains in their propensity for internal motion. Our results suggest that PDI flexibility enables it to interact with many different partner molecules of widely different sizes and shapes, and highlights considerable similarities of yPDI and mammalian PDI. Proteins 2016; 84:1776–1785. © 2016 Wiley Periodicals, Inc.

## INTRODUCTION

Proteins show internal mobility over a wide range of time‐ and length‐scales, from the rapid local motions that define the conformational entropy of a given protein conformation (<ns), to the functionally significant, concerted, slower motions (>ms) of loops or whole domains that interconvert different conformations.^1^, ^2^ The quantitative exploration of mobility across these vast timescales is important in order to understand the function of proteins, but remains a major challenge—both for proteins in isolation and in response to interactions with partner proteins and other molecules.

**Figure 1:**
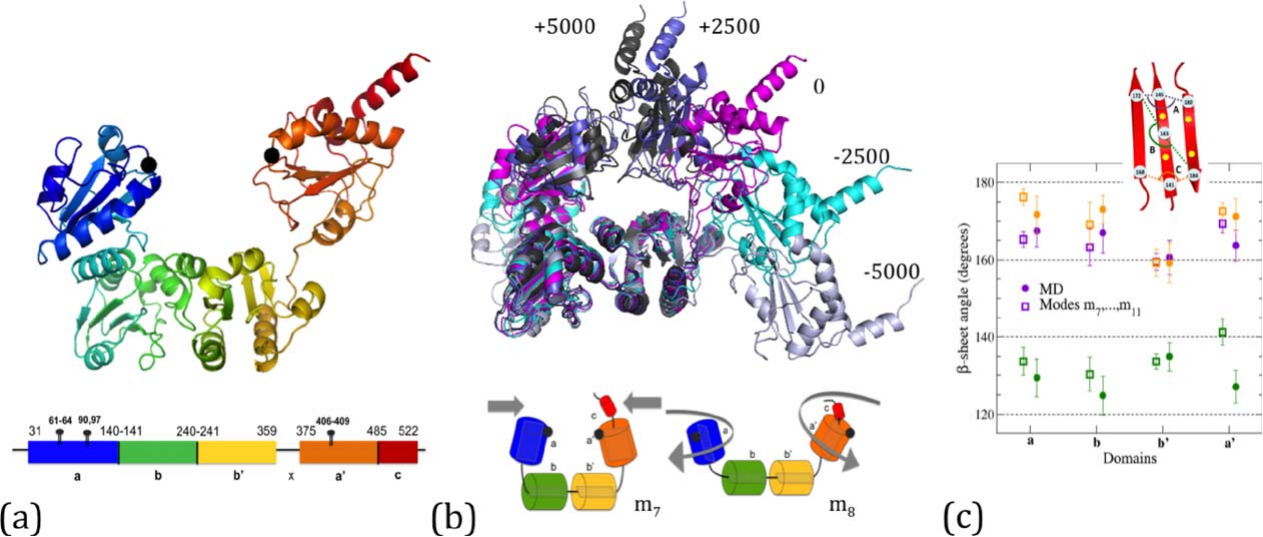
The structure of yPDI (2B5E) and its interdomain motion (a) Schematic ribbon diagram of the tertiary structure of yPDI colored from N‐terminus to C‐terminus with the **a** domain predominantly blue, the **b** domain green, the **b**′ domain yellow, and the **a**′ domain orange. The C‐terminal is shown in red and the *α*‐carbons of the active site cysteine residues 61 and 406 are shown as black spheres. The flexible **x** region is given between domains **b**′ and **a**′ in light orange. Domains **a**, **b**′, and **a**′ are situated in the same spatial plane but domain **b** (green) is displaced away from the reader. The thick line at the bottom shows the domain organization of yPDI based on the crystal structure with the four domains (**a**‐**b**‐**b**′‐**a**′), the flexible loop **x** connecting domains **b**′ and **a**′ and the C‐terminal tail **c**. Cysteine residues are shown as stalks and numbered. (b) Snapshots of conformational change for yPDI moving along mode *m*_7_. The figure shows the overlap of conformers 0, ±2500, ±5000, aligned on domains **b** and **b**′. At the bottom, we give a cartoon representation of conformational motion in normal modes *m*_7_ and *m*_8_. The planes in each domain and the two black circles schematically indicate the *β*‐sheets and the two active sites. (**c**) Averages for the relative *β*‐sheet angles in all four domains as computed from the 30 ns MD (closed symbols) and also the flexibility analysis (open symbols, averaged over modes *m*_7_, …, *m*_11_). The three different colors indicate the three measured angles for the residue triples as given in the schematic, for example, for the **b** domain with residue numbers as shown.

One of the large proteins studied intensely is protein disulfide isomerase (PDI), an abundant catalyst of oxidative protein folding in the endoplasmic reticulum of secretory cells (Fig. 1). PDI is essential for the accurate and efficient co‐ and posttranslational folding of secreted and cell‐surface proteins including medically important classes of protein such as antibodies, cytokines, digestive enzymes, blood‐clotting factors, and so forth; indeed, manipulation of PDI levels is a successful strategy used in industry for increasing the yields of high‐value recombinant disulfide‐bonded proteins.^3^ PDI comprises four domains, each of which has the conserved thioredoxin‐fold conformation (trx‐fold), arranged in sequence **a**‐**b**‐**b**′‐**x**‐**a**′‐**c**, where **a** and **a**′ are redox‐active trx‐fold domains, **b** and **b**′ are structurally similar domains lacking redox activity, **x** is an extended linker and **c** is a highly acidic C‐terminal extension^4^ [refer to Fig. 1(a)]. The function of PDI within the cell involves successive interactions with a range of diverse partner proteins and protein substrates^5^, ^6^, ^7^, ^8^ which implies that PDI is a dynamic molecule, a conclusion supported by the long delay between its discovery in the 1960s and the first determination of a crystal structure of a full‐length multidomain PDI in 2006.^9^ There is good experimental evidence for PDI as a conformationally dynamic protein whose flexibility underlies its function; studies on mammalian PDI, and also on PDI from the fungus Humicola insolens, indicate that there is extensive relative movement of the **a**′ and **b**′ domains, facilitated by the **x**‐linker.^10^, ^11^, ^12^, ^13^, ^14^, ^15^, ^16^, ^17^ By contrast, crystallization of yeast (*Saccharomyces cerevisiae*) PDI (yPDI) at different temperatures produced two crystal structures which differ markedly in the relative orientation of the **a** and **b** domains.^9^, ^18^ However, simple comparison of alternative X‐ray‐derived conformations cannot give a full picture of the dynamics of this large multidomain protein, and an exhaustive analysis by all‐atom molecular dynamics (MD) would be prohibitive in terms of computational resource. Here, we use a recently introduced rapid method^19^ for characterizing the main features of protein motion (see “Materials and Methods” section) which is based on flexibility analysis and can predict the possible mobility of a large protein such as PDI in a few computer hours. We show that the method is particularly useful in generating viable starting conformers for subsequent short MD runs, combining the speed of the flexibility analysis with the predictive power of MD.

In this article, we have two purposes: (i) to explore and describe in quantitative detail the motions of PDI and (ii) to compare our predictions to the diverse experimental data on PDI flexibility. Our results give quantitative predictions of interdomain mobility^5^, ^6^ and include a new, much more closed structure with short active‐site distance. In addition, we find motion which can connect the two known crystal structures of yPDI.^9^, ^18^ These results suggest that PDI flexibility enables it to interact with many different partner molecules of widely different sizes and shapes, and contradicts suspicions that yeast and mammalian PDI might differ significantly in properties.

## MATERIALS AND METHODS

We use a recent protein flexibility modeling approach,^19^ combining methods for deconstructing a protein structure into a network of rigid and flexible units (First)^20^ with a method that explores the elastic modes of motion of this network,^21^, ^22^, ^23^, ^24^, ^25^ and a geometric modeling of flexible motion (Froda).^26^, ^27^ The usefulness of this approach has recently been shown in a variety of systems^28^, ^29^, ^30^, ^31^, ^32^, ^33^ (see the starting sections of Supporting Information for more details.). Methods similar in spirit, exploring flexible motion using geometric simulations biased along “easy” directions, have also been implemented using Frodan^34^ and NMSim.^35^, ^36^, ^37^ We have performed our analysis through multiple (2000–5000) conformational steps starting from the high‐resolution crystal structure (pdb: 2B5E, resolution 2.4 Å), representing the full structure after every 100 steps; hence, the analysis is always based on 10 trajectories (one in each direction for each of the five most important normal modes) where each trajectory comprises 20–50 all‐atom structures in the form of PDB files. We emphasize that these trajectories do not represent actual *stochastic motion* in a thermal bath as a function of time, but rather the possibility of motion along the most relevant low‐frequency elastic modes. Each trajectory leads to a gradual shift of the protein from the starting structure, as measured by C_*α*_‐RMSD between original and derived structures, and this shift may reach an asymptote, where no further motion is possible along the initial vector, as a result of steric constraints.^19^ Energies associated with such a trajectory for bonds, angles, electrostatics, and so forth can be estimated and shown to be consistent and physically plausible. See Supporting Information for more details.

As a consistency check of our flexibility approach, we have also undertaken short, all‐atom MD simulations of the intramolecular motion of yPDI at 300 K, employing the Amber9 suite of programs^38^ with ff03 force field^39^ and parm99 parameters. Explicit solvent MD simulations are carried out on the 2B5E crystal structure of yPDI (for 30 ns) and on a “closed” structure of PDI generated from the geometric simulations as described earlier (for 10 ns). The MD simulations are performed in aqueous medium using the TIP3P water model. The solvation box is 12 Å from the farthest atom along any axis. Na^+^ ions have been added to neutralize the net charges on yPDI using the tleap module in Amber9. The MD simulations are performed under NPT conditions using the Berendsen thermostat and periodic boundary conditions. Particle Mesh Ewald summation is used for long‐range electrostatics with van der Waals cut‐off of 10 Å. The pressure and temperature relaxations are set to 0.5 ps^−1^. SHAKE constraints are applied to all bonds involving H atoms. A time step of 2 fs is employed with the integration algorithm and the structures are stored every 1 ps.

We use a variety of quantitative structural measures to describe the intra‐ and interdomain motion in yPDI as observed in the flexibility and MD simulations. To indicate the scale of the relative motion of domains in our simulations, we measure the distance *d_cc_* between C_*α*_ atoms of active site residues located in domains **a** (Cys61 in the full‐length sequence) and **a**′ (Cys406), respectively. This distance is 27 Å in 2B5E. In addition to *d_cc_*, we construct the following convenient measures of *interdomain* motion. Each of the **a**, **b**, **b**′, **a**′ domains contains a central *β*‐sheet, characteristic of the trx‐fold. The orientation of the domain can be represented by a vector normal to a central part of the *β*‐sheet. The variation of this vector between different *β*‐sheets can be described by interdomain *tilt* and *twist* angles. The normal vectors on each sheet are generated by selecting the C_*α*_ atoms of four “central” residues, namely alternating central residues in each of the adjacent antiparallel strands *β*_2_ and *β*_4_ (by PDI numbering; these are strands 1 and 3 of the trx‐fold). These four atoms define a quadrilateral and from its two diagonals we construct a normal vector $\vec{n}$. When we now consider two adjacent domains in the yPDI structure—labeled as domains 1 and 2, with central positions ${\vec{r}}_{1},\;{\vec{r}}_{2}$ and plane normals ${\vec{n}}_{1},\;{\vec{n}}_{2}$—we can define the *tilt* angle *θ* in the range 0 to 180 degrees as $\cos(\theta )={\vec{n}}_{1}\cdot {\vec{n}}_{2}$. The *dihedral twist δ*, with range from −180 to + 180 degrees, is obtained by constructing an interplane vector ${\vec{r}}_{12}={\vec{r}}_{2}-{\vec{r}}_{1}$ and considering the dihedral *δ* between the plane containing ${\vec{n}}_{1},\;{\vec{r}}_{12}$ and the plane containing ${\vec{n}}_{2},\;{\vec{r}}_{12}$ (see Supporting Information).

To describe the *intradomain* motion, we generate the *pseudodihedral ξ_i_* 40, 41, 42 for residue *i* at C_*α*_‐atom position ${\vec{r}}_{i}$. We consider the interresidue pseudo‐bonds ${\vec{q}}_{i-1},{\vec{q}}_{i},{\vec{q}}_{i+1}$ defined such that ${\vec{q}}_{i}={\vec{r}}_{i+1}-{\vec{r}}_{i}$ for all residues *i* along the protein main chain (except for the first residue in the sequence and for the last two residues). The dihedral angle *ξ_i_* formed by ${\vec{q}}_{i-1}$ and ${\vec{q}}_{i+1}$ about the axis of ${\vec{q}}_{i}$ then characterizes the orientation of the residues and their variation and flexibility. When the protein main chain is in a fully extended (*β*‐strand) state, *ξ_i_* is near zero ( $\cos(\xi_{i})\approx 1$). When the main chain is in a coiled or turned conformation (*α*‐helix or *β*‐hairpin), *ξ_i_* has magnitude above 90°, and $\cos(\xi_{i})$ is negative, typically around −0.7 for *α*‐helices. We extract $\cos(\xi_{i})$ for each residue *i* and each conformer generated during a motion. We then use the root‐mean‐square‐deviation of all such $\cos(\xi_{i})$ values as our measure of flexibility at residue *i*.

## RESULTS

### Characterization of yPDI flexibility highlights relative motion of domains

The starting point for our approach to mobility simulation of full‐length yPDI is the high‐resolution structure (pdb: 2B5E), which is used to generate a representation of the molecule as a set of rigid clusters and flexible linkers. The four‐domain structure thus obtained via First [cp. Fig. 1(a)] captures the aforementioned conventional **a**, **b**, **b**′, **a**′ domains as well as the **x**‐linker and the **c** terminal.^43^, ^44^, ^1^ We then apply the method for simulating protein motion^19^ to the high‐resolution structure to move the structure along vectors that describe the low‐frequency modes of motion of the protein (see Supporting Information Fig. S1). Here, we have focused on normal modes 7–11 (*m*_7_–*m*_11_) which are the five lowest‐frequency nontrivial normal modes (*m*_1_–*m*_6_ describe trivial rotations and translations). We find that the motions are primarily motions of **a** and (especially) **a**′ domains relative to a **b**‐**b**′ base. Figure 1(b) summarizes the trajectory along mode *m*_7_ by showing superimposed structures representing the conformers computed at steps 0, ±2500 and ±5000. The central domains **b** and **b**′ form an essentially invariant base, while in comparison the N‐ and C‐terminal domains **a** and **a**′ show coordinated movement toward and away from each other, relative to their positions in the crystal structure; movement in the positive sense closes the structure, while that in the negative sense opens it. Figure 1(b) also represents this motion in cartoon terms and shows a similar representation of yPDI motion along the next lowest‐frequency mode *m*_8_ where the motions are essentially rotations of domains **a** and **a**′ around intradomain axes. For the next low‐frequency modes *m*_9_–*m*_11_, the motion can be roughly characterized as comprising combinations of hinge motions and rotations of domains (see also Supporting Information Fig. S2).

We next generated a relatively short molecular MD trajectory >30 ns (consuming >36,000 CPU hours) and interrogated the trajectory in a number of ways to compare it with the outputs from the computationally significantly less demanding flexibility approach. Both the flexibility and MD simulations generate trajectories comprising a series of all‐atom protein structures and hence both can be subjected to the same analyses. Initially, we asked whether we could confirm that the major features of motion were motions of whole domains. To test if the domains remained essentially intact as structural units, we obtained measures of *β*‐sheet geometry for each domain and asked how constant these measures remained through the simulations of motion. For each domain, we selected a number of C_*α*_ atoms to define the sheet and extracted angles between sets of three atoms as measures of sheet geometry. We then derived the mean and standard deviation of these angles through the trajectories of motion obtained both by MD simulation and flexibility analysis. Figure 1(c) plots these data for each domain and shows (i) that each angle measure shows a very narrow variation through the motion simulations (standard deviations are ±2–3° for the flexibility simulations and ±5–6° for the MD simulations) and (ii) that there is very close agreement between both types of simulation in the mean angles observed. These data confirm that the core *β*‐sheets of each domain retain essentially constant geometry throughout both sets of simulation (see also Supporting Information Fig. S3).

Let us emphasize that, here and in the following, the aim of the relatively short MD runs is just to provide a consistency check on the flexibility results. Clearly, we would expect that the full range of motion found in, for example, Figure 1, by the flexibility approach will be recovered by longer MD runs as well.

### Distinctive character of interdomain motion predicted by twists and tilts of β‐sheets

**Figure 2:**
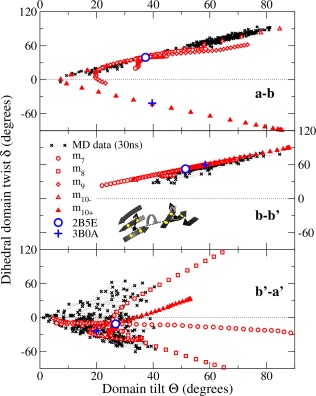
Characterization of interdomain mobility of yPDI. The panels show the tilt and dihedral twist motion (see “Materials and Methods” section) between neighboring domain pairs **a**‐**b**, **b**‐**b**′ and **b**′‐**a**′. The black crosses indicate results from the MD run, whereas the red symbols denote the modes *m*_7_, *m*_8_, *m*_9_, and *m*_10_ (*m*_11_ not shown for clarity). For *m*_10_, we additionally distinguish between the directions of motion *m*_10–_ and *m*_10+_, highlighting how the positive direction leads to the “high‐temperature” crystal structure 3BOA. The blue symbols denote tilt and twist for the two indicated crystal structures. The thin gray lines connect the MD results as they are computed along the MD trajectory. The horizontal dotted lines indicate a twist *δ* = 0. The inset graphics in the central panel indicates schematically the geometric interpretation of tilt and twist angles based on a quadrilateral anchored at four residues (yellow spots) on each *β*‐sheet (see also Supporting Information). [Color figure can be viewed at wileyonlinelibrary.com]

To provide a quantitative account of the large‐scale interdomain motion of this multidomain protein, we have represented each domain as a plane based on its core *β*‐sheet and calculated the relative orientations of these planes, tilt (*θ*) and dihedral twist (*δ*), through simulations of motion. Figure 2 presents a twist/tilt plot for each adjacent domain pair and allows a direct comparison of the predictions from flexibility analysis and MD simulations. The upper panel represents the **a**‐**b** domain pair and shows that the MD simulation explores a considerable range around the 2B5E starting structure, but that all orientations fall close to a single line in twist/tilt space. Flexibility analyses in modes *m*_7_–*m*_9_ predict a similar pattern of motion, but modes *m*_10_ (and *m*_11_, which we do not show for clarity) begin to explore other regions of twist/tilt space. In particular, motion along the positive direction of *m*_10_, converts the relative orientation of the **a** and **b** domains into precisely that found in the alternative “high‐temperature” crystal structure of yPDI (pdb: 3BOA, resolution 3.7 Å); this structure lies far away from the region explored in the MD simulation. The correspondence to the 3BOA structure provides experimental crystallographic evidence that our identification of the character of motion that is possible at the **a**‐**b** domain interface is correct. As the 2B5E and 3BOA structures are connected by flexible motion, we suggest that they are not separated by a major structural transition. Rather, they are both members of one wide‐ranging flexible ensemble in solution, from which the two crystal structures are selected by slight differences in crystallization conditions. A very similar phenomenon was recently identified in the domain orientation of ERp27.^45^

The central panel shows comparable data for the **b**‐**b**′ domain pair and indicates that MD and flexibility both explore the same limited region of twist/tilt space but with the flexibility simulation extending slightly further away from the orientations defined by the crystal structures. The data in the lower panel refer to the **b**′‐**a**′ domain pair and show different characteristics. The MD simulation covers an extended area of twist/tilt space while the normal mode trajectories form various paths through this area and also extend well beyond it; both simulations predict very extensive relative motion of this domain pair (see also Supporting Information Fig. S4).

**Figure 3:**
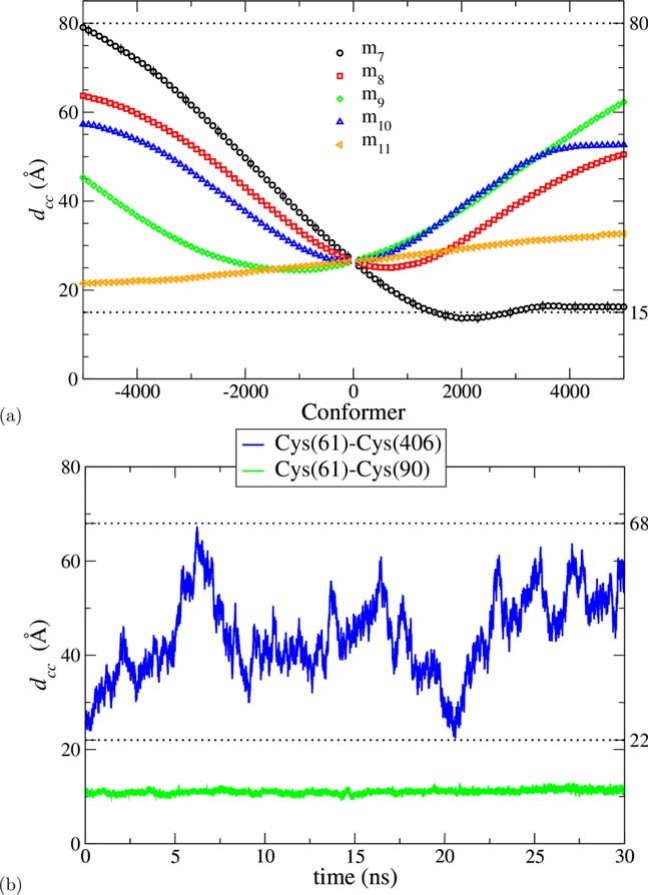
Interdomain motion generates large change in distance between active sites. (**a**) Distance *d_cc_* between the C_*α*_ atoms of cysteine residues Cys61 and Cys406 as a function of conformer generated as the structure moves along the normal modes. Error bars indicate error‐of‐mean for five different starting conditions (shown for *m*_7_ and every 5th symbol only). (**b**) Evolution of *d_cc_* between cysteine pairs for the 30 ns MD simulation. The horizontal dotted lines indicate the observed range of distances for the Cys61–Cys406 pair. [Color figure can be viewed at wileyonlinelibrary.com]

Figure 3(a) shows the variation of the distance *d_cc_* between active site residues located in domains **a** (Cys61) and **a**′ (Cys406). It demonstrates that movement along *m*_7_ allows *d_cc_* to vary freely from 15 to 80 Å due to the coordinated hinge‐like motion of domains **a** and **a**′ relative to **b**‐**b**′. Figure 3(a) also shows the less striking evolution of the intersite distance through the other normal mode trajectories, reflecting the more complex motions in these modes. Figure 3(b) shows that during the 30 ns MD simulation, the intersite distance increases from its initial value and then varies widely through the trajectory, ranging up to 68 Å (see also Supporting Information Figs. S5 and S6).

### Intradomain motion and flexibility

**Figure 4:**
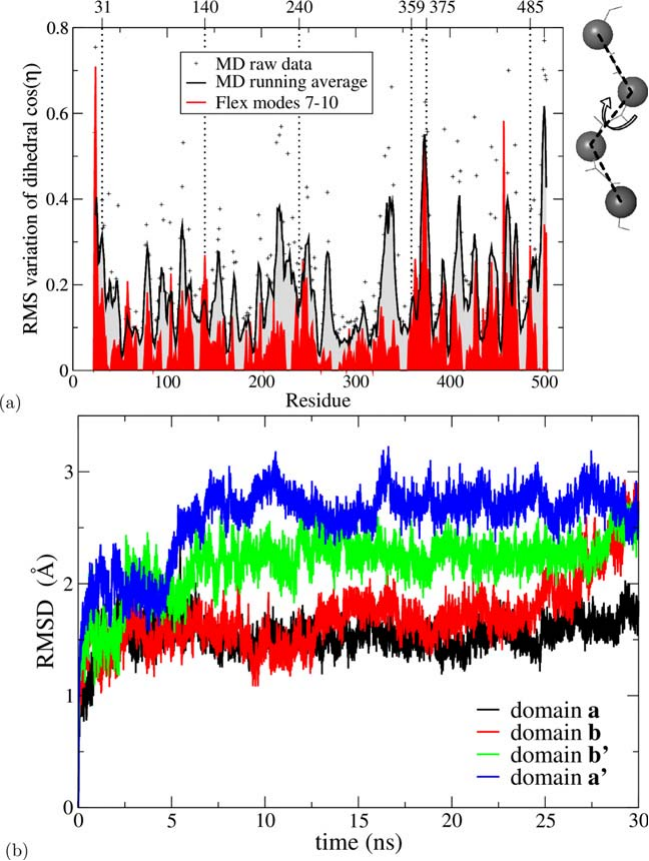
Domains show clear differences in intradomain mobility (**a**) Pseudodihedral RMS variation plot versus sequence from long MD run (raw data and running average >5 residues) and for the flexibility analysis averaged over *m*_7_, …, *m*_10_ at *E*_cut_ = −2 kcal/mol. The domain boundaries are marked with dotted vertical lines. The schematic on the right indicates the definition of the pseudodihedral along the protein backbone. (**b**) C_*α*_‐RMSD as a function of simulation time for yPDI domains. The values are obtained by overlapping each domain from the initial crystal structure with itself from the conformers generated during a 30 ns MD simulation. [Color figure can be viewed at wileyonlinelibrary.com]

To focus more on local, intradomain motion, we have determined the pseudodihedral angle *ξ* 40, 42 at each residue for each structure computed in modes *m*_7_–*m*_10_ and then derived the RMS variation of cos⁡ξ as a measure of the net flexibility at each residue. As shown in Figure 4(a), this analysis highlights as regions of greatest flexibility the N‐ and C‐termini and the **x**‐linker, with local maxima also shown at the **a**‐**b** and **b**‐**b**′ domain boundaries. This confirms that the major flexibility of the protein arises from the relative motion of domains but it also indicates that there is considerable intradomain motion. Figure 4(a) also displays the variation of pseudodihedral angle *ξ*, as derived from the MD analysis. MD detects much more local and higher frequency motion and hence it is not surprising that the MD‐derived plot shows much greater scatter. Taking a running average >5 residues smooths some of this and again shows that the most marked motion is at the termini and at the **x**‐linker; motion at the other domain boundaries is not more marked than at several intradomain sites. Interestingly, both methods indicate much greater intradomain motion within the **a**′ domain than within the other domains.

To obtain an alternative measure of the extent of intradomain motion through our simulations, we monitored the evolution of the MD trajectory >30 ns in terms of the C_*α*_‐Root mean square deviation between each of the domains and the structure of that domain in the initial full‐length protein [Fig. 4(b)]. It is apparent that each domain initially deviates to some extent from its original structure in the crystal, but that for the period from 5 to 25 ns, these differences are approximately stable, with domain **a**′ showing the greatest RMSD and domains **a** and **b** showing the smallest. What also emerges from Figure 4(b) is that the MD analysis over this period does not explore the full potential for motion as discussed earlier. In the period from 25 ns onwards, domain **b** (in contrast to the other domains) begins a further phase of motion, which considerably increases its RMSD from the starting structure. For comparison, we analyzed the change in RMSD for each domain through the flexibility trajectories of modes *m*_7_–*m*_11_ (see also Supporting Information Fig. S7).

### Stability of an extreme “closed” structure

**Figure 5:**
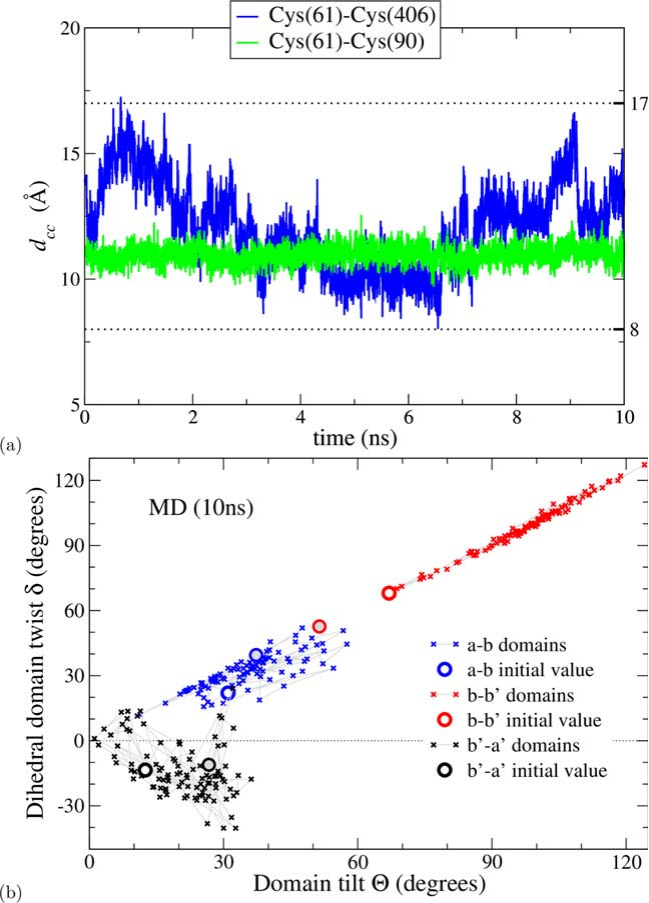
MD analysis of extreme structure generated by flexibility shows that it is stable. MD analysis was performed >10 ns starting from the “extreme closed” structure generated by flexibility analysis (cp. Fig. 1). (**a**) Evolution of distances between Cys residue pairs. (**b**) Tilt/twist analysis; the initial tilt/twist values (open circles) are from the “extreme closed” structure while the values corresponding to the crystal structure of 2B5E are shown as gray‐shaded circles. [Color figure can be viewed at wileyonlinelibrary.com]

Our analysis of motion along normal mode *m*_7_ suggested that the molecule was able both to “open” and “close” relative to the starting structure, generating values for the intersite distance in the range 15–80 Å [Fig. 3(a)]. To test whether such closed structures were artifacts of our flexibility approach, a short MD simulation (10 ns) was performed on a “closed” structure to assess its physical plausibility. We took the atomic coordinates of a “closed” structure representing the extreme of positive motion along *m*_7_ and used it as the starting point for the MD simulation. Figure 5(a) shows the MD trajectory >10 ns starting from this extreme “closed” structure, expressed in terms of the intersite distance. The molecule moves gradually to explore structures in which the distance between the active site residues 61 and 406 lies in the range from 8 to 17 Å. The intradomain distance between cysteines at residues 61 and 90 remains constant during the simulation as in Figure 3(b). Two conclusions are very clear from this simulation, (i) the closed structure is physically plausible as it is not immediately abandoned in the first few ns of the MD simulation, and an ensemble of “closed” conformations are explored by MD simulations, (ii) the original 30 ns MD simulation did not fully explore the conformational space available to yPDI, as it never generated any structure comparable to those found in this 10 ns simulation (as judged by the value of the intersite distance). These conclusions are confirmed by the data in Figure 5(b), which represent the trajectories from the 10 ns MD simulation in terms of twist/tilt plots. Hence, the flexibility analysis clearly finds energetically plausible interdomain orientations, which do not simply regress from the “closed” structure toward the orientations found in the crystal structure. It is known in the literature^46^, ^47^ that short multiple MD simulations, with different starting conformations, can explore the conformational space better than a single long equilibrium simulation. Our results from two MD simulations reiterate this point. More importantly, we have shown that the flexibility approach can complement MD simulations by quickly providing such a set of realistic, different starting points.

## DISCUSSION

The main feature of PDI molecular motion predicted by both flexibility and MD approaches is that the redox‐active **a** and **a**′ domains show considerable freedom of motion with respect to a relatively fixed base provided by the **b** and **b**′ domains. This motion includes both rotations and hinge‐like opening and closing about the **a**‐**b** and **b**′‐**a**′ hinges. These findings on the scope for flexibility of yPDI are valuable in two respects: (i) they suggest that differences in the literature data on the mobilities of PDIs from yeast and other sources can easily be reconciled and (ii) they provide insight into PDI's cellular function as a catalyst of oxidative protein folding and its interactions with substrates and partner proteins.

A substantial body of work in human PDI and its fragments in solution, using techniques such as partial proteolysis, intrinsic fluorescence and high‐field NMR^10^, ^11^, ^12^, ^13^, ^14^ has shown that the **x** linker region is a site of substantial flexibility and that its motion relative to the adjacent **b**′ domain can modulate access to the major noncovalent ligand binding site on **b**′ and alter the orientation of this domain relative to its neighboring catalytic **a**′ domain. A major change in relative orientation of these two domains was also inferred from NMR, X‐ray crystallographic, and Small‐angle X‐ray scattering studies on the reduced and oxidized states of the fungus *H. insolens* 15, 16 and has now been observed directly by X‐ray crystallography for human PDI.^12^ Very recently, a crosslinking/mass spectrometry study on human PDI in solution found numerous crosslinks could form between the **a** and **a**′ domains, including between residues that are >50 Å apart in the crystal structure, providing dramatic evidence for extensive interdomain mobility in solution giving rise to structures much more compact than those observed in crystals.^17^ Similarly, a 300 ns large‐scale MD analysis starting from reduced and oxidized human PDI crystal structures, detected formation of compact conformations including ones in which the active sites in **a** and **a**′ domains were in close proximity.^48^ By contrast, work on yPDI has provided valuable insights, based almost entirely on X‐ray crystal structures, with very little comparative work on mobility in solution. The X‐ray work, based on yPDI crystals grown at different temperatures, has provided two structures which differ very little in the relative orientation of the **b**′ and **a**′ domains, but show clear differences in the N‐terminal half of the protein, in the relative orientation of the **a** and **b** domains.^9^, ^18^ The simulations reported here are based on the higher resolution yeast structure, derived from crystals grown at 4°C. They show that there is extensive motion of both **a** and **a**′ domains, with the motion of the **a**′ domain being more marked, but that the mode 10 trajectory involves a rotation of the **a** domain relative to the **b** domain which results in the orientation found in the higher temperature crystal structure. Our modeling of yPDI flexibility indicates that yPDI shows extensive mobility both in the **a**‐**b** and in the **b**′‐**x**′‐**a**′ regions, comparable to that observed experimentally in solution for other PDIs, but that the nonstatistical sampling of conformations provided by the crystallization has highlighted only the **a**‐**b** motion.

The extensive flexibility observed in our work also throws light on the most striking of PDI's functional properties, namely its wide substrate range—its ability to interact with diverse newly synthesized proteins destined for secretion. These substrates for oxidative protein folding (i.e., newly synthesized proteins in the reduced unfolded state, plus partly oxidized and partly refolded intermediates) can differ widely in size, shape, and charge. Using the small model protein, basic pancreatic trypsin inhibitor, we recently showed that PDI interacts with this folding substrate at every stage along its oxidative folding pathway.^49^ As part of its cellular function, PDI also interacts with a variety of partner proteins resident in the endoplasmic reticulum that together perform the complete cellular process of oxidative protein folding.^4^, ^6^, ^7^, ^8^ These partners, which differ widely in size and shape, include the oxidase Ero1 with which PDI undergoes a redox interaction and the holoenzyme prolyl‐4‐hydroxylase in which PDI plays a chaperone role. So the key facts about PDI interactions are (i) that there is an enormous range and variety of proteins that interact with PDI as substrates or partners and (ii) that the main site of ligand binding to PDI is a hydrophobic area on the **b**′ domain facing in to the volume defined by the **abb**′**xa**′ “horseshoe.”^4^, ^5^, ^11^ However, there are no high resolution structures of PDI bound to a substrate or partner protein. The structure of the oxidase Ero1 has been determined and, in this case a model of the human PDI/human Ero1alpha contact has been generated by docking simulation.^50^ But in most cases the structure of the partner is not known. Our work shows clearly that PDI has considerable scope to alter the relative distances and orientations of three key functional sites, namely the redox active sites in the **a** and **a**′ domains and the site on the **b**′ domain where protein ligands bind noncovalently. We have used the distance between redox active sites as the major indicator of this flexibility, but could also have used the volume enclosed within the PDI “horseshoe” or the distances between other functional sites. It is reasonable to infer that such a promiscuous protein as PDI will exploit its flexibility to interact with its wide range of partners that differ considerably in size and shape. Furthermore, the core of the ligand‐binding site is an exposed hydrophobic region on the **b**′ domain located on the inner surface of the “horseshoe.”^11^ Within the **b**′ domain, we observe extensive intradomain flexibility [Fig. 4(b)] and this could permit a wide variety of ligand proteins to dock effectively with this site.

## CONCLUSIONS

The wide‐ranging and disparate experimental data that indicate the extensive and functionally significant flexible motion of PDI have now been combined with structurally detailed simulations which confirm the character and extent of this flexibility. Our results show that there is interdomain flexibility at every interdomain junction but showing very different characteristics, that is, extensive freedom to tilt and twist at **b**′‐**a**′, constrained to a specific twist mode at **a**‐**b**, and with no freedom to twist at **b**‐**b**′. There is also intradomain flexibility and clear differences between the domains in their tendency for such internal motion. The extent of the interdomain flexibility is such that the two active sites can approach much more closely than is found in crystal structures. Indeed, we find that hinge motion to bring these sites into proximity is the lowest energy normal mode of motion of the protein. Furthermore, the flexibility predicted for yPDI (2B5E) includes the other known conformation (3BOA) and is consistent with the mobility observed experimentally for mammalian PDI.

Our flexibility‐based method provides quantitative measures, such as distances and angle variations, to be tested in the future by, for example, crosslinking and FRET experiments. In addition, as demonstrated here, the generated conformers can serve as starting points for more in‐depth MD runs, combining the remarkable computational economy of the flexibility approach with the biologically more detailed MD dynamics. Such a combination should be most interesting, for example, when applied to a study of the whole family of PDI^5^ as determined since the 2006 publication of yPDI (2B5E).

## Supporting Information

More details on the flexibility analysis and the MD approach, as well as the structural measures used, can be found in the Supporting Information.

## Supporting Citations

References 51, 52, 53 appear in the Supporting Information.

## Supporting information

Supporting Information 1

## Footnotes

1 Results for Structural classification of Proteins and Class Architecture Topology Homology analyses can be readily obtained from the PDB entry for 2B5E.
