# Supplementary material for: The flexibility and dynamics of protein disulfide isomerase
Source: Proteins. 2016 Oct 1;84(12):1776–85. doi: 10.1002/prot.25159 (PMC5111589; doi:10.1002/prot.25159)
Supplement: Supplementary file 1 — Supporting Information 1 [file PROT-84-1776-s001.pdf]

SUPPORTING INFORMATION

The dynamics and flexibility of protein disulphide-isomerase (PDI): simulations predict experimentally-observed domain motions

Rudolf A Römer, Stephen A Wells, J Emilio Jimenez-Roldan, Moitrayee Bhattacharyya, Saraswathi Vishweshwara and Robert B Freedman

RIGID CLUSTER AND NORMAL MODE ANALYSIS OF YEAST PDI

The starting point for our approach to mobility simulation of full-length yeast PDI (yPDI) is the high-resolution structure (pdb: 2B5E), which is used to generate a representation of the molecule as a set of rigid clusters and flexible linkers. In Figure 1 we show the structure schematically both as a ribbon diagram and as a linear representation, highlighting its organization as 4 distinct domains with an extended linker between domains **b'** and **a'**. The rigid cluster analysis includes bonds as constraints but is dependent on setting a 'cut-off' energy parameter (E_{cut}) to include strong H-bonds and exclude weaker ones (as inferred from the high-resolution structure). Figure S1 illustrates the effect of varying the value of E_{cut}. Inclusion of all H-bonds leads to a representation of PDI as a single rigid cluster. Exclusion of the weakest bonds gives a model that shows the molecule as four distinct rigid clusters corresponding closely to the structurally-defined domains. Further stepwise exclusion of weaker bonds then leads either to fragmentation of each cluster into a series of smaller clusters (see e.g. domain **a'**) or to maintenance of the domain as a single cluster, but with specific regions becoming flexible and hence excluded from the cluster (see e.g. domain **a**). The rigidity analysis brings out the greater relative rigidity of the four domains and the flexibility of the interdomain linker regions. We emphasise, however, that even once the domains become flexible, they are still well constrained by an internal network of noncovalent interactions in the folding core of each domain. Previous experience [30, 45] has shown that biologically significance flexible motion is best modelled at E_{cut} values around -2 to -3 kcal/mol. We have explored flexible motion at cutoffs of -2, -3, and -4 kcal/mol, obtaining very similar results in each case. We report results in detail for $E_{\text{cut}} = -2$ kcal/mol. The normal modes are calculated using a standard elastic network approach [23],

i.e. without non-harmonic force fields. We restrict our presentation to the first 5 non-trivial modes $m_7, \ldots, m_{11}$ as these should be sufficient to capture the most important aspects of the collective motion for yPDI [19, 21]. Additional modes could also easily be explored, and we have analysed trajectories for modes m_{12}–m_{16} (data not shown) but we find that the lowest 5 modes capture the majority of the accessible motion and provide a good overall picture with considerable structural detail. In many cases the mode trajectory is eventually limited by steric constraints, providing an amplitude limit on the motion. In Figure S2, we give a cartoon representation of the five modes m_7–m_{11}. Mode m_7 is the opening and closing mode as discussed in detail in the main text. Mode m_8 corresponds to large rotational motion of domain **a'** and smaller rotation of **a**. Seen from above, the rotation is anti-clockwise for both **a** and **a'**. Mode m_9 is a simultaneous counter rotation of domains **a** (anti-clock) and **a'** (clockwise) of similar amplitude while mode m_{10} is the closing/opening movement of m_7 for **a'** combined with an anti-clockwise rotation in m_{10}. Last, mode m_{11} is a rolling motion of domains **b** and **b'** coupled with a rotation of the **a** and **a'** domains that is similar to m_9.

MORE DETAILS ON THE MOBILITY ANALYSIS

The measures of β-sheet angles as shown in Figure 1c represent averages over the 30 ns MD. Figure S3 shows the full evolution of these β-sheet angles through the MD simulation, demonstrating the limited range of values found for each angle and the lack of trend over time. This result indicates the remarkable stability of the β-sheet geometry and hence of the integrity of domains during motion in yPDI.

An alternative analysis of the relative motion of domains can be made by selecting a single central point to represent each domain and then calculating through each trajectory the four-domain dihedral angle (**a-b** vs. **b'-a'**) and the two interdomain angles **a-b-b'** and **b-b'-a'**. These measures capture the relative overall positions of domains, but not their relative orientations. As presented in Figure S5, this analysis shows that MD simulations and flexibility simulations make similar predictions for the range covered by the **b-b'-a'** interdomain angle and come close to predicting the relative domain positions found in the 'alternative' crystal structure of yPDI. But the simulations differ in respect of the range of **a-b-b'** angle and the range of four-domain dihedral angle found; in both cases, the flexibility simulations explore a wider range.

COMPARING MD AND THE FLEXIBILITY APPROACH

Empirical-potential MD and rapid simulations of flexible motion have quite different conceptual bases. MD makes use of a detailed force-field which defines a high-dimensional energy landscape. Physically acceptable conformations of the protein correspond to low-energy regions of this landscape, while unacceptable conformations have high energy. The MD simulation moves across the energy landscape by numerical integration of classical equations of motion. In contrast, the flexibility simulations place constraints on local bonding geometry and steric exclusion while neglecting longer-range interactions. This has the effect of simplifying and flattening the low-energy regions of the MD energy landscape, removing a large number of small energetic or kinetic barriers, while still forbidding access to the high-energy regions with unacceptable steric overlaps or distortions of bonding geometry. The two approaches are complementary in that MD provides data on high-frequency local motion while the flexibility approach allows a more rapid exploration of the full range of interdomain motion.The flexibility simulations can pass rapidly between different regions in conformational space, which are in principle accessible to MD simulations but in practice would only be explored on very long timescales. We note that there are of course several other methods including replica exchange MD (REMD), umbrella sampling, etc. [51] to sample a wider conformational space.

As discussed in the Methods section, our flexibility analysis does not directly map onto motion in a thermal bath or free energy landscape. Nevertheless, we can roughly estimate the energy variation involved during the Froda dynamics by constructing structural energy estimates for each obtained conformer using, e.g., the GROMOS96 force field within Swiss-PdbViewer [53]. Energetically prohibited conformers would correspond to rapidly increasing energy values while a stable conformer trajectory should be consistent with a flat energy profile. In Figure S4 we show the energies for every 500th Froda conformation for representative modes m_7 and m_{10}. In each case, we present the full set of 6 possible energy terms as well as the total energy. The bond and angle energies are almost constant. There is a very slight increase compared to the input as the trajectory progresses, but clearly the template system in Froda is working effectively to constrain the local bonding geometry as intended. The electrostatic term is also almost constant. This suggests that it is dominated by local polar interactions which in turn are well constrained as they are identified as con-

straints by FIRST and maintained by FRODA. The torsion term increases gradually during trajectories. This is because the dihedral angles are free to vary in a FRODA trajectory, so they will be moving into and across the higher-energy portions of the dihedral potential. The scale of this effect is small compared to the total energy. Non-bonded interactions dominate the total energy and effectively control its variation. Interestingly, in all trajectories, the non-bonded energy actually *decreases* over the early stages of the trajectory (see conformer 500 in each case) by substantial amounts of around 10% of the total energy. We attribute this to the gradual resolution of steric clashes present in the initial 2B5E structure. Later, as the trajectories progress, the energies corresponding to the non-bonded terms becomes less negative and hence the conformer is less stable. The worst destabilisation we see here reaches about 20% compared to the B2E5 structure. Nevertheless, these results suggest that (i) there is no rapid increase in energy due to wrong conformers being formed and (ii), while some increase in energy is observed, it is of a similar order of magnitude as the energy gain from the B2E5 structure when the FRODA dynamics allows for relaxation of steric clashes. These results support the validity of our flexibility approach.

Our rapid flexibility analysis requires only a few CPU hours to generate many trajectories for this large (c. 500 residues) protein in full atomistic detail, providing the basis for a close analysis of molecular motion. Given the fundamental conceptual and operational differences between MD and the rapid flexibility approach — and the difference of several orders of magnitude ($> 10^4$) in computer time that MD requires — their predictions are strikingly congruent in many respects. The methods provide very similar predictions of the range of interdomain orientations explored (Figure 2), the variation of inter-active-site distance that is accessible (Figure 3), and the extent to which the individual domains show intradomain flexibility (Figure 4). The MD approach provides a far more detailed picture of local motion (Figure 4a) but does not explore some very large amplitude domain motions, which are detected by the flexibility approach and are supported by experimental data. One example is the very large change in the **a-b** interdomain twist angle (Figure 2a) predicted by motion along mode 10, which generates precisely the relative orientation of these domains found in an alternative crystal structure [18]. A further example is given by flexible motion along mode 7, which can generate 'closed' structures with a much shorter inter-active-site distance (ca. 15Å) than that found in either crystal structure. Experimental data indicate that these sites can be cross-linked by bifunctional chemical reagents with maximum span

16Å [52] highlighting the extent to which the 'horseshoe' structure of PDI can close in solution. Furthermore, although such structures were not generated in the initial 30 ns MD simulation, such closed structures are rather stable (Figure 5) over a 10 ns MD simulation, with limited amplitude fluctuations in domain orientation and inter-active-site distance.

MORE ON THE USE OF THE STRUCTURAL MEASURES

Pseudodihedral RMS:

The conformation of the protein main chain can be almost completely specified by giving the two Ramachandran angles (φ, ψ) for each residue. These define the geometry of the C-N-C_α-C and N-C_α-C-N variable dihedrals around the C_α atom. The variation of the Ramachandran angles in the course of a simulation is then a measure of the flexibility of the protein main chain. However, much of the information present in the Ramachandran angles can be captured using one number per residue rather than two, by considering a pseudodihedral measure defined by the C_α atoms of four successive residues [40–42]. The variation of this backbone pseudodihedral is, in turn, a convenient measure of protein flexibility.

Obviously we are concerned less with the absolute value of ξ_i, but more with the variation in ξ_i during motion. Our approach to describing the flexibility of the protein is as follows. For each structure generated during simulations of motion, we extract $\cos(\xi_i)$ for each residue i. We then find the mean and variance of $\cos(\xi_i)$ over the course of the simulation for each residue. The root-mean-square-deviation of $\cos(\xi_i)$ is our measure of flexibility.

Tilt and dihedral twist:

Let us identify the atoms at the corners of a quadrilateral as a, b, c, d in cyclic order (cp. the schematic in Figure 2). They have position vectors $\vec{r}_\text{a}$, $\vec{r}_\text{b}$, $\vec{r}_\text{c}$, $\vec{r}_\text{d}$. We obtain a central position $\vec{r}$ for the plane as $\vec{r} = (\vec{r}_\text{a} + \vec{r}_\text{b} + \vec{r}_\text{c} + \vec{r}_\text{d})/4$. Vectors representing the diagonals of the quadrilateral are $\vec{r}_\text{ac} = \vec{r}_\text{c} - \vec{r}_\text{a}$ and $\vec{r}_\text{bd} = \vec{r}_\text{d} - \vec{r}_\text{b}$ and $\vec{n} = \vec{r}_\text{ac} \times \vec{r}_\text{bd}$ is the unit normal vector $\vec{n}$ parallel to the cross product of the two.

We now consider two adjacent domains in the yPDI structure, which we label as domains 1 and 2, with central positions $\vec{r}_1$, $\vec{r}_2$ and plane normals $\vec{n}_1$, $\vec{n}_2$. An interdomain tilt angle

θ in the range 0 to 180 degrees is obtained from $\cos(\theta) = \vec{n}_1 \cdot \vec{n}_2$. An interdomain dihedral twist δ in the range -180 to $+180$ degrees is obtained by constructing an interplane vector $\vec{r}_{12} = \vec{r}_2 - \vec{r}_1$ and considering the dihedral δ between the plane containing $\vec{n}_1$, $\vec{r}_{12}$ and the plane containing $\vec{n}_2$, $\vec{r}_{12}$. Two natural motions for adjacent domains are (i) a "towards-and-away" tilting motion, in which θ varies substantially with little variation in δ, and (ii) an "axial twist" motion of rotation about the interplane vector, generating a co-variation of θ and δ. Motions of this kind are visible in our tilt-twist plots as horizontal and diagonal trajectories.

Tilt and twist values were extracted for the input structures, for the structures generated in the simulations of flexible motion, and for conformers of the MD trajectory, to describe three relative domain orientations: **a-b**, **b-b'**, and **b'-a'**. The residues selected to represent plane orientations in yPDI are as follows: In domain **a**, residues LEU 108, ILE 110, LEU 53, and GLU 55; in domain **b**, residues LEU 202, ILE 204, ILE 163 and GLN 165; in domain **b'**, residues PHE 314, ILE 316, GLY 260 and LEU 262; last, in domain **a'**, residues ILE 453, LEU 455, LEU 398 and LEU 400. Note that we use next-nearest-neighbour rather than nearest-neighbour residues in each strand, in order to prevent the pleating of the β-sheet affecting our results.

ADDITIONAL ANALYSIS OF THE INTER- AND INTRADOMAIN MOTION

Figure 3 shows how flexibility (Figure 3a) and MD (Figure 3b) explore the interdomain motion. Extending this analysis of flexible motion in terms of the distance d_{cc} between active sites, Supporting figure S6 plots the evolution of d_{cc} through flexible motion in modes m_7–m_{11} for a range of values of the parameter E_{cut}. Lower values of E_{cut} correspond to greater limitations on flexible motion and, as expected, they lead to more restricted motion, as measured by this parameter.

The histogram of the MD inter-site distances (Figure S7) is derived from the data in Figure 3b and confirms the wide spread of distances with the most common distance being close to 40 Å. Although this interdomain distance varies widely through the MD simulation, intradomain distances (exemplified by the distance between the active-site residue Cys61 and a buried residue within the same domain (Cys90)) remain essentially constant (Figure 3b) and shows a very tight distribution around 11 Å (Figure S7).

Figure S8 shows that in the majority of modes, the intradomain C_α-RMSD is greatest for the **a’** domain and smallest for **a** and **b** domains. This trend in intradomain motion is also evident in the analysis of variation of backbone pseudodihedral angles (Figure 4a) where both MD simulation and flexibility analysis show greatest variation in the **a’** domain.

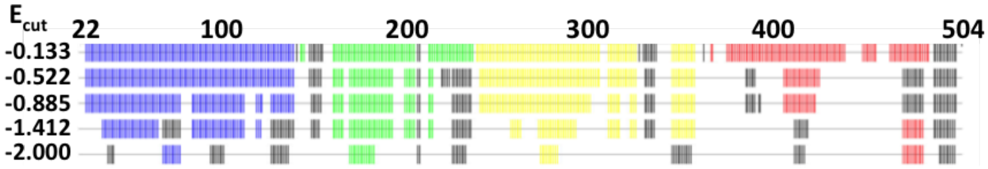

FIG. S1. Rigid cluster decomposition graph. The horizontal axis represents the protein backbone and the vertical axis the energy $E_{\rm cut}$. The residues belonging to rigid clusters are colored as in Figure 1 whereas the flexible regions are shown as horizontal thin black lines.

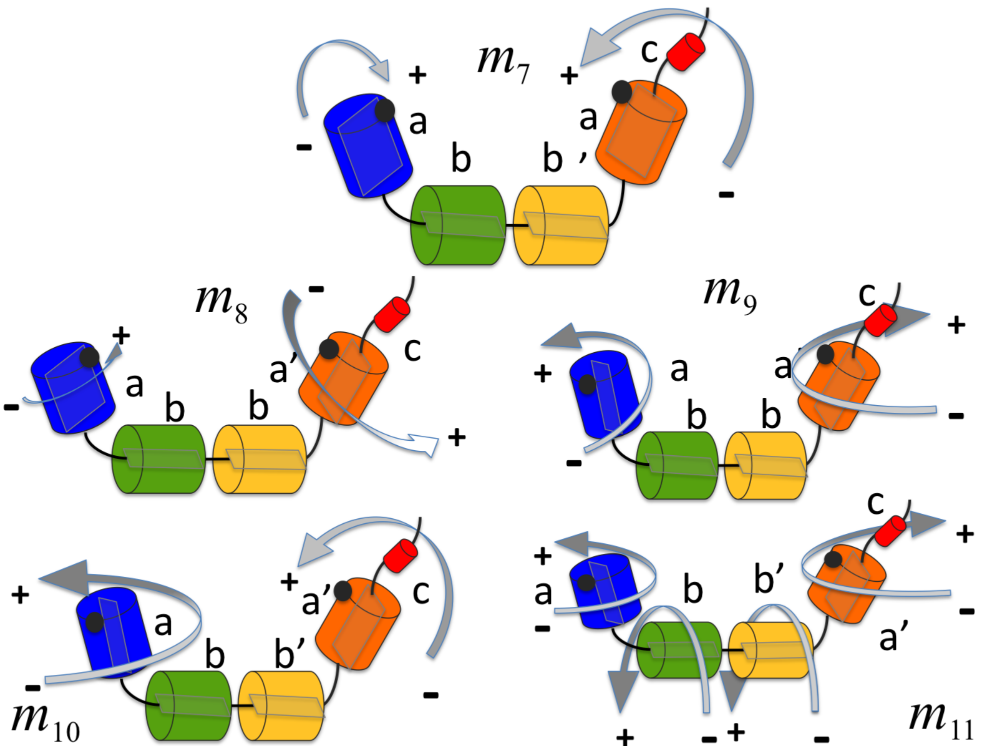

FIG. S2. Cartoon representation of conformational motion for all five normal modes m_7, m_8, m_9, m_{10} and m_{11}. The planes in each domain and the two black circles schematically indicate the β-sheets and the two active sites. The 4 large barrels denote the domains **a** (blue), **b** (green), **b'** (yellow) and **a'** (red) while the smaller barrel denotes the **c** tail (dark red). Arrows indicate movement according to the normal modes in positive (+) and negative (−) directions as discussed in the main text. The shading of the arrows becomes lighter to indicate out of the plane movement, whereas darker arrows indicate movement into the plane.

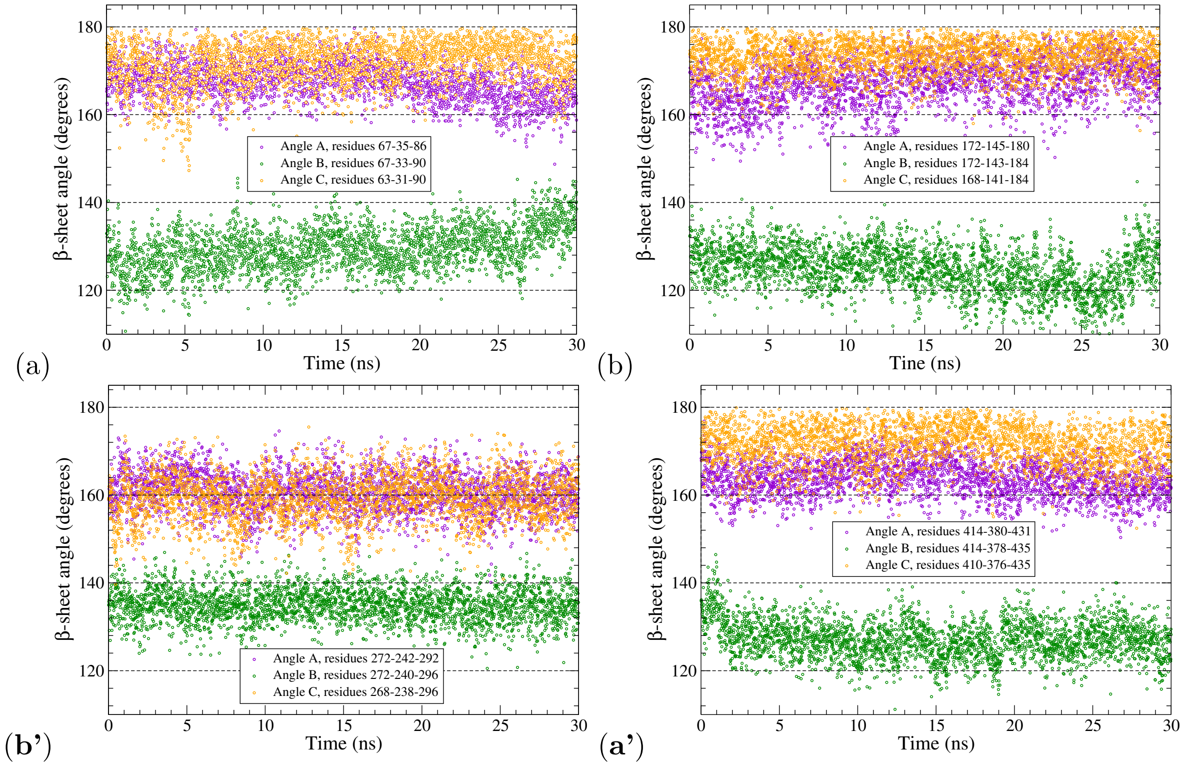

FIG. S3. The β-sheet angles remain stable along the 30 ns MD trajectory. The three colours correspond to the three angles defined on the indicated residues for each β-sheet. See also Figure 1c.

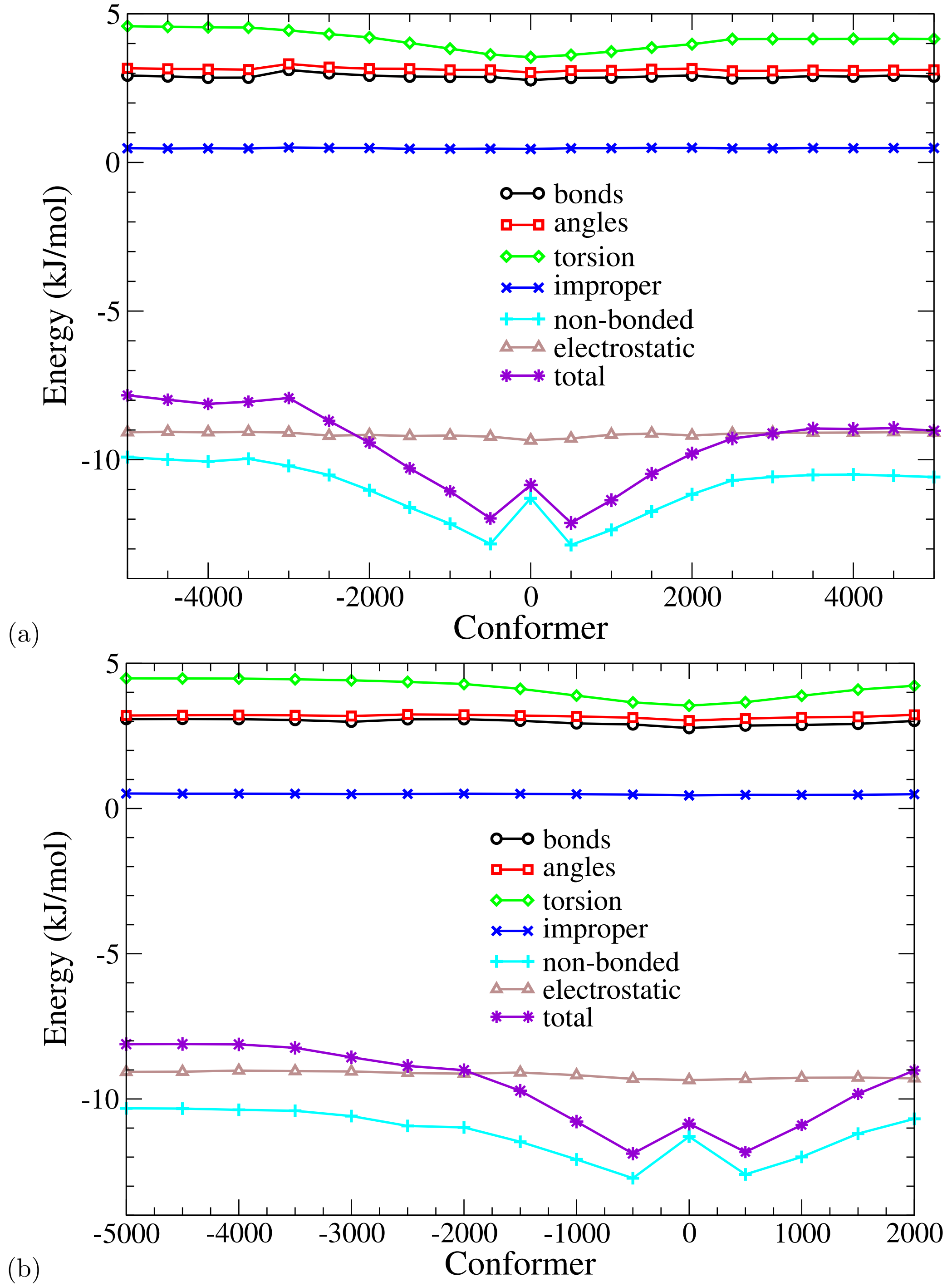

FIG. S4. Energies associated with bonds (○), angles (□), torsion (◊), improper (×), non-bonded (+), and electrostatic (Δ) interactions [53] as a function of conformer generated as in Figure 3(a) starting from 2B5E. The total energy is indicated by a star symbol (∗). The panel (a) corresponds to mode m_7 while (b) displays the behaviour of mode m_{10}. Steric clashes dominate beyond conformer 2000 (not shown for m_{10}).

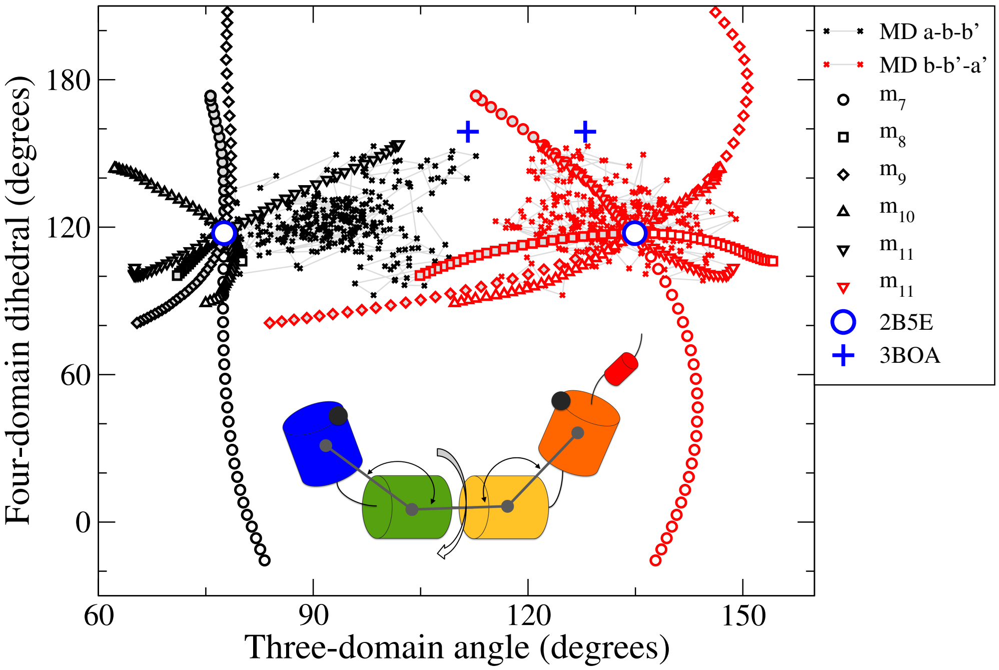

FIG. S5. Four-domain analysis of PDI flexibility. The black data points (left of figure) show the variation of the four-domain dihedral angle (broad open arrow in cartoon) with respect to the **a-b-b'** three domain angle (left thin arrow in cartoon, cp. Figure 1), while the red data points (right of figure) show the variation of the four-domain dihedral with the **b-b'-a'** three domain angle (right thin arrow in cartoon). The open symbols show negative modes $m_{7-}, \ldots, m_{11-}$, the filled symbols are for positive modes $m_{7+}, \ldots, m_{11+}$. The blue circle indicates the starting values for structure 2B5E while the blue cross shows the alternative 3BOA structure. The site chosen to represent each domain is the C_α of a centrally located residue within the β-sheet: the selected residues are ALA 86 (domain **a**), VAL 191 (domain **b**), VAL 291 (domain **b'**) and ALA 433 (**a'**). Inset: Schematic view of the four-domain dihedral arrangement (white arrow) and the 2 three-domain angles (black arrows).

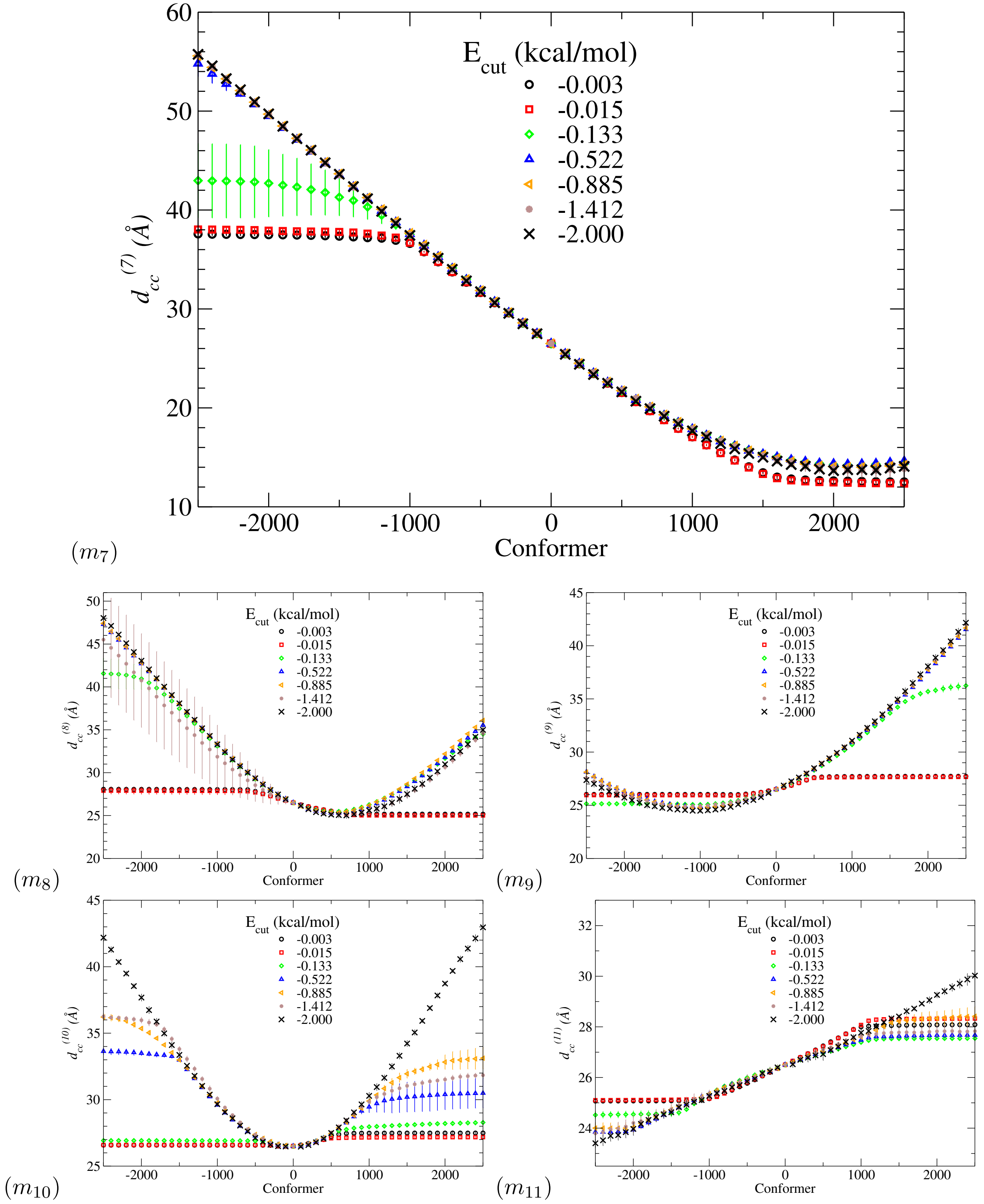

FIG. S6. Distance d_{cc} between the cysteine active sites in the **a** and **a'** domains for various modes (m_7 (top), $m_8, \ldots, m_{11}$) and E_{cut} values. The d_{cc} distances correspond to the intercysteine distance of the conformers obtained as the protein is projected along (a) mode m_7, (b) mode m_8, (c) mode m_9,(d) mode m_{10} and (e) mode m_{11} along closing (positive) and opening (negative) directions.

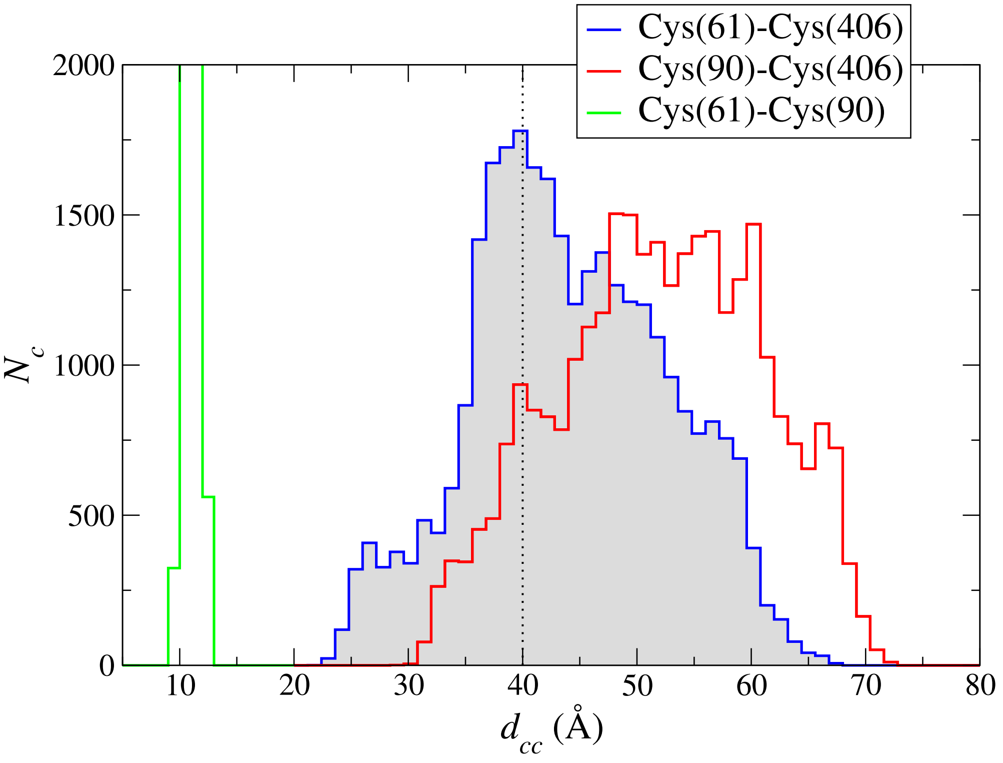

FIG. S7. Histogram of inter-cysteine distances generated through the 30 ns MD simulation. The vertical dotted line indicates the value $d_{cc} = 40$ Å. See also Figure 3.

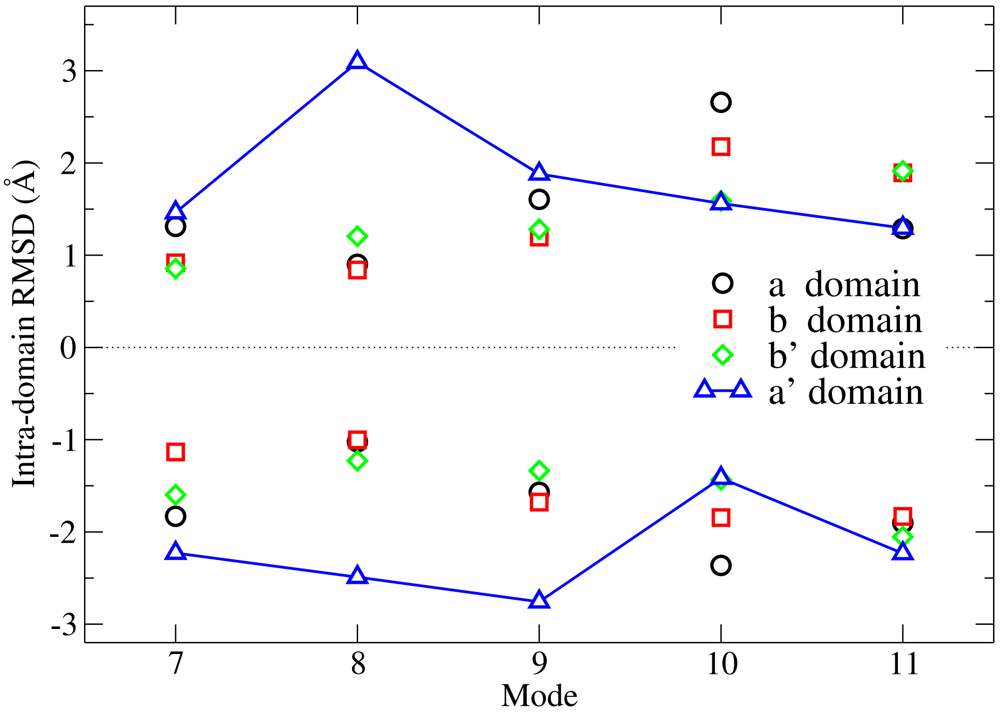

FIG. S8. Change in intradomain C_α-RMSD for modes $m_7, \ldots, m_{11}$. The line connects RMSD values for the **a'** domain, which exhibits the largest intradomain RMSD for modes 7, 8 and 9. See also Figure 4.
